# Pre- and post-hemodialysis differences in heart failure diagnosis by current heart failure guidelines in patients with end-stage renal disease

**DOI:** 10.1186/s44348-024-00003-8

**Published:** 2024-06-12

**Authors:** Bong-Joon Kim, Su-Hyun Bae, Soo-Jin Kim, Sung-Il Im, Hyunsu Kim, Jung-Ho Heo, Ho Sik Shin, Ye Na Kim, Yeonsoon Jung, Hark Rim

**Affiliations:** 1 Division of Cardiology, Department of Internal Medicine, Kosin University College of Medicine, Gospel Hospital, Busan, Korea; 2 Division of Nephrology, Department of Internal Medicine, Kosin University College of Medicine, Gospel Hospital, 262 Gamcheon-Ro, Seo-Gu, Busan, 49267 Korea; 3 Transplantation Research Institute, Kosin University College of Medicine, Busan, Korea

**Keywords:** End-stage renal disease, Hemodialysis, Heart failure, Diagnosis

## Abstract

**Background:**

Patients with end-stage renal disease (ESRD) who are on hemodialysis (HD) have reduced vascular compliance and are likely to develop heart failure (HF). In this study, we estimated the prevalence of HF pre- and post-HD in ESRD using the current guidelines.

**Methods:**

We prospectively investigated HF in ESRD patients on HD using echocardiography pre- and post-HD. We used the structural and functional abnormality criteria of the 2021 European Society of Cardiology guidelines.

**Results:**

A total of 54 patients were enrolled. The mean age was 62.6 years, and 40.1% were male. Forty-five patients (83.3%) had hypertension, 28 (51.9%) had diabetes, and 20 (37.0%) had ischemic heart disease. The mean N-terminal-pro brain natriuretic peptide BNP (NT-proBNP) level was 12,388.8 ± 2,592.2 pg/dL. The mean ideal body weight was 59.3 kg, mean hemodialysis time was 237.4 min, and mean real filtration was 2.8 kg. The mean left ventricular ejection fraction (LVEF) was 62.4%, and mean left ventricular end-diastolic diameter was 52.0 mm in pre-HD. Post-HD echocardiography showed significantly lower left atrial volume index (33.3 ± 15.9 vs. 40.6 ± 17.1, *p* = 0.030), tricuspid regurgitation jet V (2.5 ± 0.4 vs. 2.8 ± 0.4 m/s, *p* < 0.001), and right ventricular systolic pressure (32.1 ± 10.3 vs. 38.4 ± 11.6, *p* = 0.005) compared with pre-HD. There were no differences in LVEF, E/E′ ratio, or left ventricular global longitudinal strain. A total of 88.9% of pre-HD patients and 66.7% of post-HD patients had either structural or functional abnormalities in echocardiographic parameters according to recent HF guidelines (*p* = 0.007).

**Conclusions:**

Our data showed that the majority of patients undergoing hemodialysis satisfy the diagnostic criteria for HF according to current HF guidelines. Pre-HD patients had a 22.2% higher incidence in the prevalence of functional or structural abnormalities as compared with post-HD patients.

## Introduction

Mortality is high in end-stage renal disease (ESRD) patients undergoing hemodialysis (HD), and heart failure (HF) is a leading cause of mortality among these patients [1, 2]. ESRD patients with HF exhibit an approximately 2-year shorter survival rate after HD initiation compared with patients without HF [3]. There are differences between patients with ESRD and healthy people regarding hemodynamic status. In particular, many ESRD patients have left ventricular hypertrophy (LVH); previous data showed that 74% of patients with ESRD have LVH at initiation of dialysis therapy [4], and concentric hypertrophy is more common than eccentric hypertrophy in ESRD patients [5]. Features like LVH or left atrium (LA) enlargement might be an adaptive remodeling response to long-term volume or pressure overload [6]. High inter-dialytic weight gain is associated with a greater risk of cardiovascular events [7]. As cardiac remodeling progresses slowly, there are many patients who are clinically stable without symptoms of HF while undergoing hemodynamic adaptation. However, many ESRD patients satisfy the objective diagnostic criteria for HF suggested by current guidelines.

According to the 2021 European Society of Cardiology (ESC) guidelines, structural or functional abnormalities of HF should be evaluated using several echocardiographic parameters: left ventricular mass index (LVMI), relative wall thickness, left atrial volume index (LAVI), E/E′ ratio, and tricuspid regurgitation (TR) velocity at rest [8]. Several guidelines recommend using the H2FPEF score [9] or HFA-PEFF score [10] for heart failure with preserved ejection fraction (HFpEF) diagnosis, but they are too complex and difficult to apply to clinical practice. Thus, it is not easy to clearly distinguish the presence of HF in clinical practice. Furthermore, there is a discrepancy between objective parameters from guidelines and practical settings as to whether most ESRD patients undergoing HD should be included in HF diagnosis. Dialysis patients experience a volume change of up to 3–4 kg before and after HD, which can affect hemodynamic indicators. Therefore, depending on when echocardiography is performed, there may be differences in hemodynamic parameters. However, it is not easy to perform echocardiography in the euvolemic state in the clinical setting. Therefore, we aimed to evaluate the prevalence of HF diagnosis using the current guidelines before and after HD in ESRD patients.

## Methods

### Study design and population

This was a single-center, prospective study conducted at a university hospital. We enrolled ESRD patients undergoing HD between November 2022 and March 2023 at Kosin University Gospel Hospital. All patients visited the hospital on the agreed date and visited the echocardiography room immediately before dialysis. A single cardiologist conducted a medical history that included a physical examination and checked for the following symptoms and signs of HF according to the 2021 ESC guidelines [8], breathlessness, orthopnea, paroxysmal nocturnal dyspnea, and reduced exercise tolerance. After a short interview, we performed pre-HD echocardiography and sent the patients to the hemodialysis room. Immediately after dialysis, they returned to the echocardiography room and underwent post-HD echocardiography. As most of the patients underwent 4-h dialysis, the interval between pre-HD and post-HD echocardiogram was approximately 5 h. Demographic and comorbidity data were obtained from the hospital medical records.

### Echocardiography measurement

One cardiology professor (who has a specialty in echocardiography) and four sonographers performed echocardiography using two instruments (Vivid E9; GE Healthcare, Boston, MA, USA and Philips iE33; Philips Medical Systems, Bothell, WA, USA). We aimed to have the same examiner perform pre- and post-echocardiography on the same patient when possible; however, this was not always achieved. Left ventricular (LV) cavity diameter, left ventricular end-diastolic volume (LVEDV)/left ventricular end-systolic volume (LVESV), and LVMI were measured according to the criteria outlined by the American Society of Echocardiography (ASE) [11]. To assess LV diastolic function, we measured peak E-wave velocity, peak A-wave velocity, mitral valve (MV) E/A ratio, MV deceleration time, pulsed-wave tissue Doppler imaging e′ velocity, mitral E/e′, LAVI, and TR jet velocity (m/s) as recommended by the 2016 ASE/European Association of Cardiovascular Imaging guidelines [12]. Right ventricular systolic pressure (RVSP; in mmHg) was calculated from TR V_max_ using a simplified Bernoulli formula: 4 × (TR V_max_)^2^ + right atrial (RA) pressure. The RA pressure was determined according to the inferior vena cava (IVC) diameter and the presence of inspiratory collapse. The cut-off of dilated IVC was > 2.1 cm, and collapse was defined as > 50% during sniff [13]. We used the following criteria for structural or functional abnormality: LVMI ≥ 95 g/m^2^ in females, ≥ 115 g/m^2^ in males, LAVI > 34 mL/m^2^, E/e′ ratio > 14, TR jet velocity at rest > 2.8 m/s. The standard for an abnormal E/e′ ratio is different for each set of guidelines, but the value was recently set to correspond with the diastolic function standard of ASE [12, 14].

### Statistical analyses

Statistical analyses were performed using the IBM SPSS Statistics software Version 25.0 (IBM Corp., Armonk, NY, USA). Data normality was tested using the Kolmogorov–Smirnov test. Values are expressed as means (± standard deviation) for numerical variables or as numbers of participants and percentages for categorical variables. Continuous variables were compared using the Student’s t-test. Categorical data were analyzed using the χ^2^ test. A p-value < 0.05 was considered to indicate statistical significance.

## Results

A total of 54 patients were enrolled. Mean age was 62.6 years, and 40.1% of participants were male. Forty-five patients (83.3%) had HTN, 28 (51.9%) had diabetes mellitus, 20 (37.0%) had ischemic heart disease (11 of whom had percutaneous coronary intervention or coronary artery bypass graft), and 8 patients (14.8%) had atrial fibrillation (AF) (Table 1). A total of 61.1% of patients were taking renin-angiotensin system (RAS) blocking agents containing an angiotensin receptor neprilysin inhibitor (ARNI), and 61.1% were taking a β-blocker. Among the five patients taking ARNI, one patient had heart failure with reduced ejection fraction and 4 patients had HF with improved ejection fraction (EF) prior to enrollment in our study; the left ventricular ejection fraction (LVEF) was less than 40% in the previous, and the EF measured in this study was greater than 40%. The mean N-terminal-pro brain natriuretic peptide BNP (NT-proBNP) level was 12,388.8 ± 2,592.2 pg/dL. Table 2 shows the hemodynamics between HD. The mean HD time was 237.4 min, and the mean real filtration was 2.8 kg. Systolic blood pressure (BP) decreased by 13.5 mmHg, and diastolic BP decreased by 2.2 mmHg after HD.

**Table 1:** Baseline characteristics

Variable	Patients with HD (*n* = 54)
Age, mean (year)	62.6 ± 10.8
Male, n (%)	22 (40.7)
Height, cm	162.5 ± 9.0
Body weight, kg	59.6 ± 13.1
Hypertension, n (%)	45 (83.3)
Diabetes Mellitus, n (%)	28 (51.9)
Dyslipidemia, n (%)	37 (68.5)
Stroke, n (%)	6 (11.1)
History of malignancy	3 (5.6)
Ischemic heart disease	20 (37.0)
PCI or CABG	11 (20.4)
Valvular heart disease	9 (16.7)
Atrial fibrillation, n (%)	8 (14.8)
Medications	
ACEI or ARB, n (%)	28 (52.8)
ARNI, n (%)	5 (9.3)
Beta-blocker, n (%)	33 (61.1)
CCB, n (%)	26 (48.1)
Antiplatelets, n (%)	30 (55.6)
Statin, n (%)	34 (63.0)
NOAC, n (%)	8 (14.8)
Warfarin, n (%)	3 (5.6)
Anti-glycemic drugs, n (%)	23 (42.6)

All values are presented as mean SD. *PCI* percutaneous coronary intervention, *CABG* coronary artery bypass graft, *ACEI* angiotensin converting enzyme inhibitor, *ARB* angiotensin receptor blocker, *ARNI* angiotensin receptor-neprilysin inhibitor, *CCB* calcium channel blocker, *NOAC* new oral anticoagulant

**Table 2:** Hemodialysis data

Variable	Patients with HD (*n* = 54)
Mean time of hemodialysis, min	237.4 ± 16.6
Ideal body weight, kg	59.3 ± 13.9
Pre HD body weight, kg	62.1 ± 13.2
Post HD bodyweight, kg	59.4 ± 13.0
Real filtration, kg	2.8 ± 1.0
Pre HD SBP, mmHg	145.0 ± 21.0
Pre HD DBP, mmHg	74.0 ± 12.4
Pre HD HR, bpm/min	72.1 ± 9.5
Post HD SBP, mmHg	131.5 ± 23.0
Post HD DBP, mmHg	71.8 ± 12.9
Post HD HR, bpm/min	76.6 ± 14.9

*HD*, hemodialysis, *SBP* systolic blood pressure, *DBP* diastolic blood pressure, *HR* heart rate

Echocardiography parameters in pre- and post-dialysis states are shown in Table 3. The mean LVEF was 62.4% on pre-HD. Compared to pre-HD echocardiography, post-HD echocardiography showed significantly reduced left ventricular end-diastolic diameter (47.1 ± 6.3 vs. 34.3 ± 6.8 mm, *p* < 0.001), LVESD (31.0 ± 5.4 vs. 34.3 ± 6.8 mm, *p* = 0.006), LVEDV (104.2 ± 34.0 vs. 132.9 ± 41.8 mL, *p* < 0.001), and LVESV (39.2 ± 17.4 vs. 53.1 ± 28.1 mL, *p* = 0.003). Among the parameters of HF criteria, post-HD echocardiography showed significantly lower LAVI (33.3 ± 15.9 vs. 40.6 ± 17.1, *p* = 0.030), TR jet V (2.5 ± 0.4 vs. 2.8 ± 0.4 m/s, *p* < 0.001), and RVSP (32.1 ± 10.3 vs. 38.4 ± 11.6, *p* = 0.005) compared with pre-HD. There was no difference in LVEF (pre vs. post, 62.4 ± 8.5 vs. 63.5 ± 7.2%, *p* = 0.495), E/e′ ratio (16.2 ± 6.2 vs. 14.9 ± 6.5, *p* = 0.299), or left ventricular global longitudinal strain (LV GLS; − 15.9 ± 5.0% vs. − 15.6 ± 5.0%, *p* = 0.721).

**Table 3:** Echocardiography parameters in pre and post dialysis

	Pre-Hemodialysis	Post-Hemodialysis	*p*-value
LV EF (M-mode), %	62.4 ± 8.5	63.5 ± 7.2	0.495
LVEDD, mm	52.0 ± 7.2	47.1 ± 6.3	<0.001
LVESD, mm	34.3 ± 6.8	31.0 ± 5.4	0.006
LVEDV, mL	132.9 ± 41.8	104.2 ± 34.0	<0.001
LVESD, mL	53.1 ± 28.1	39.2 ± 17.4	0.003
IVSTd, mm	11.5 ± 2.1	11.5 ± 2.1	0.888
PWTd, mm	10.1 ± 1.6	9.7 ± 1.5	0.277
LV mass, g	221.0 ±72.6	185.5 ± 64.3	0.010
LVMI, g/m^2^	135.1 ± 46.6	114.9 ± 39.7	0.021
LA diameter, mm	39.8 ± 6.6	37.6 ± 6.2	0.085
Aorta diameter, mm	30.4 ± 4.4	32.0 ± 4.6	0.089
LAVI	40.6 ± 17.1	33.3 ± 15.9	0.030
E velocity, cm/sec	91.0 ± 23.3	72.0 ± 26.3	<0.001
A velocity, cm/sec	91.1 ± 22.5	104.7 ± 48.3	0.078
E/A ratio	1.0 ± 0.3	0.8 ± 0.3	0.001
E/E’	16.2 ± 6.2	14.9 ± 6.5	0.299
TR jet V, m/s	2.8 ± 0.4	2.5 ± 0.4	<0.001
RVSP, mmHg	38.4 ± 11.6	32.1 ± 10.3	0.005
IVC diameter, cm	1.5 ± 0.4	1.3 ± 0.4	0.013
LVEF by Simpson method on 4CH, %	60.7 ± 7.8	62.8 ± 7.1	0.146
LVEF by Simpson method on 2CH, %	61.5 ± 7.6	62.8 ± 7.2	0.367
LV GLS, %	-15.9 ± 5.0	-15.6 ± 5.0	0.721

All values are presented as the mean ± SD. *ADHF* acute decompensated heart failure, *LVEF* left ventricular ejection fraction, *LVEDD* left ventricular end-diastolic diameter, *LVESD* left ventricular end-systolic diameter, *LVEDV* left ventricular end-diastolic volume, *LVESD* lefr ventricular end-systolic volume, *IVSTd* diastolic interventricular septal wall thickness dimension, *PWTd* diastolic posterior wall thickness dimension, *LVMI* left ventricular mass index, *LA* left atrium, *E* peak early diastolic mitral filling velocity, A peak late diastolic mitral filling velocity, E’, early diastolic mitral annular velocity, *TR jet V* maximal tricuspid regurgitation velocity, *RVSP* right ventricle systolic pressure ventricular mass index, *PWTd* diastolic posterior wall thickness dimension, *RVSP* right ventricular systolic pressure, TR jet V maximal tricuspid regurgitation velocity

**Fig. 1:**
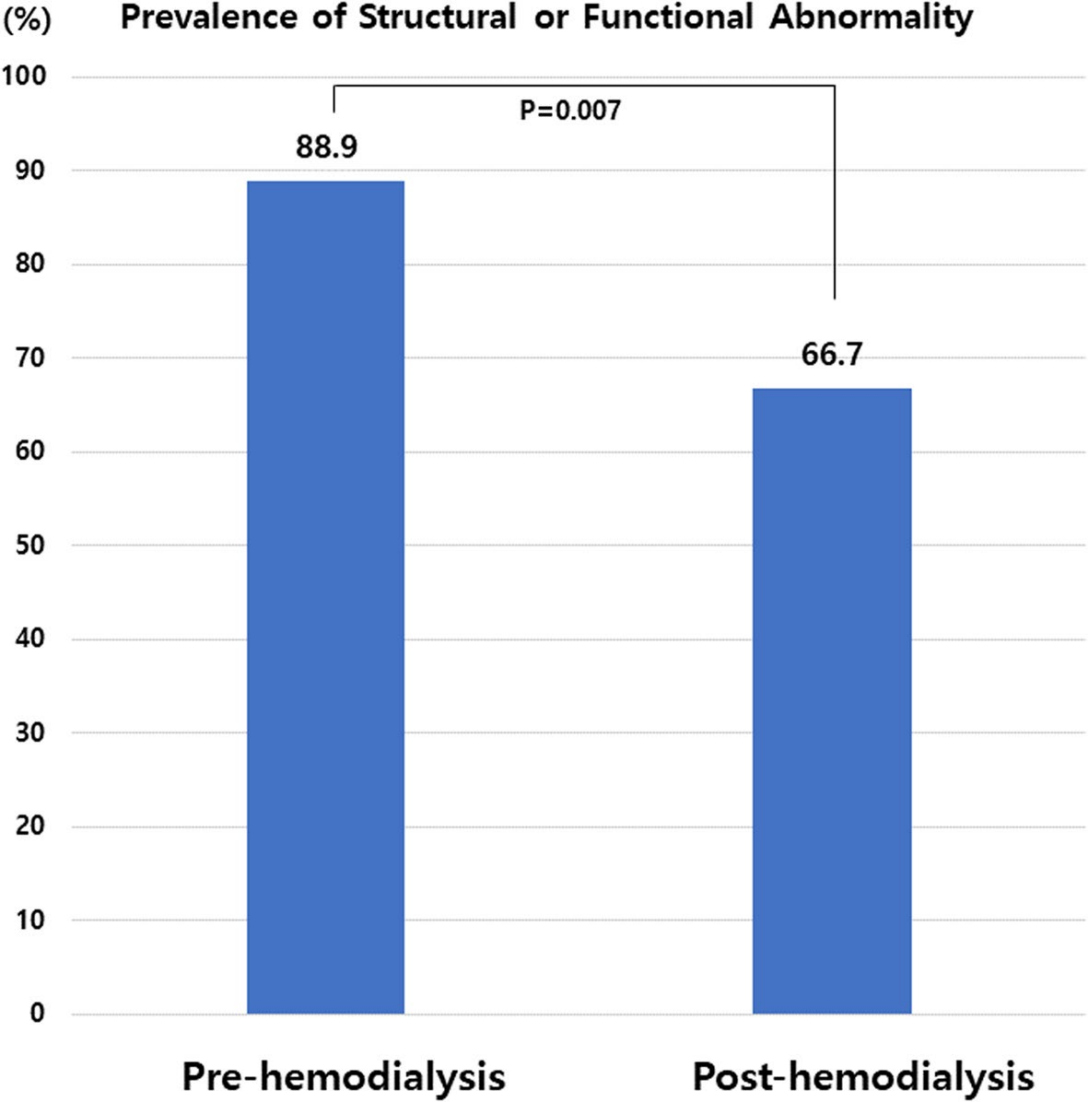
Prevalence of structural or functional abnormalities in pre- and post-hemodialysis

**Fig. 2:**
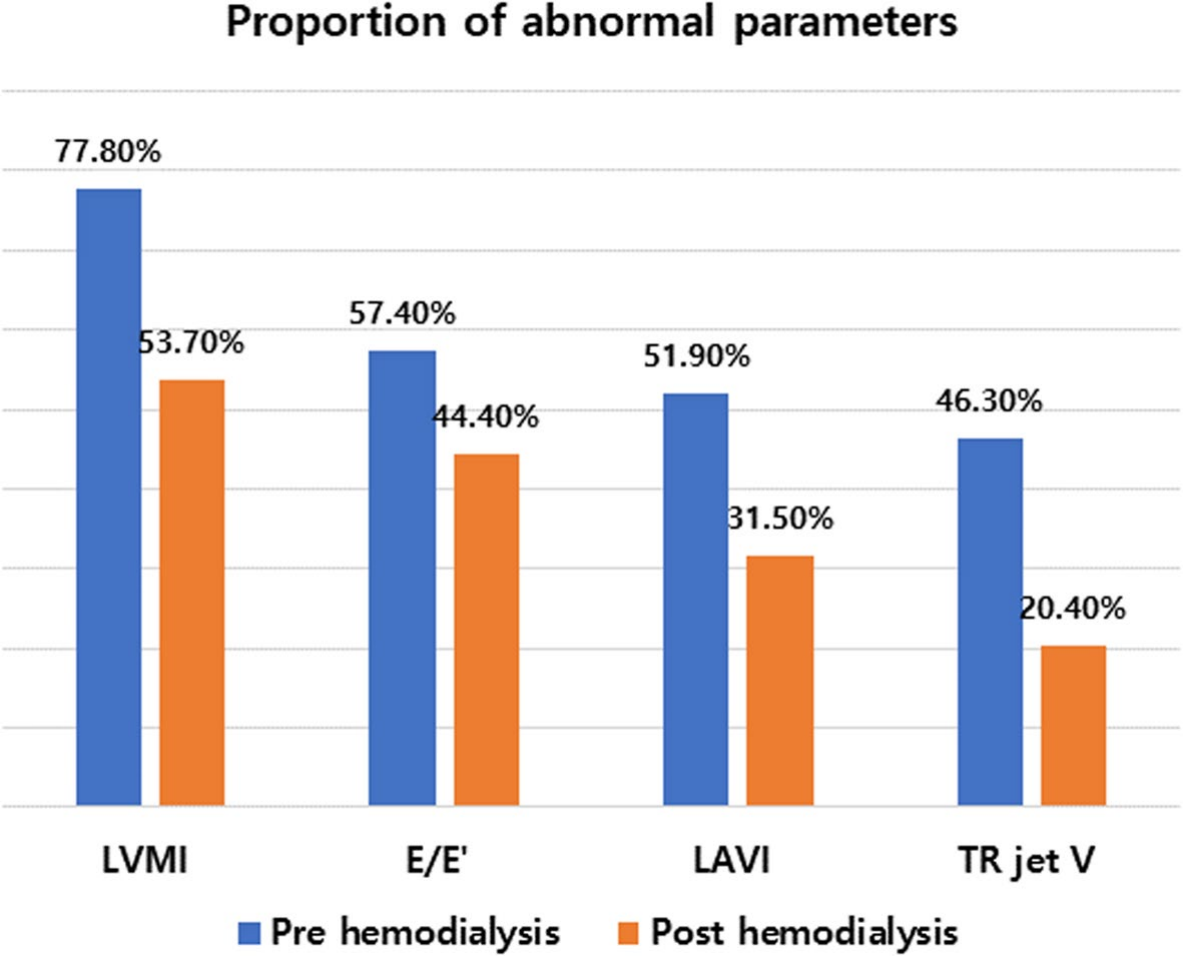
Proportion of abnormal structural or functional parameters. E: peak early diastolic mitral filling velocity, E′: early diastolic mitral annular velocity, LAVI: left atrial volume index, LVMI: left ventricular mass index, TR jet V: maximal tricuspid regurgitation velocity

**Fig. 3:**
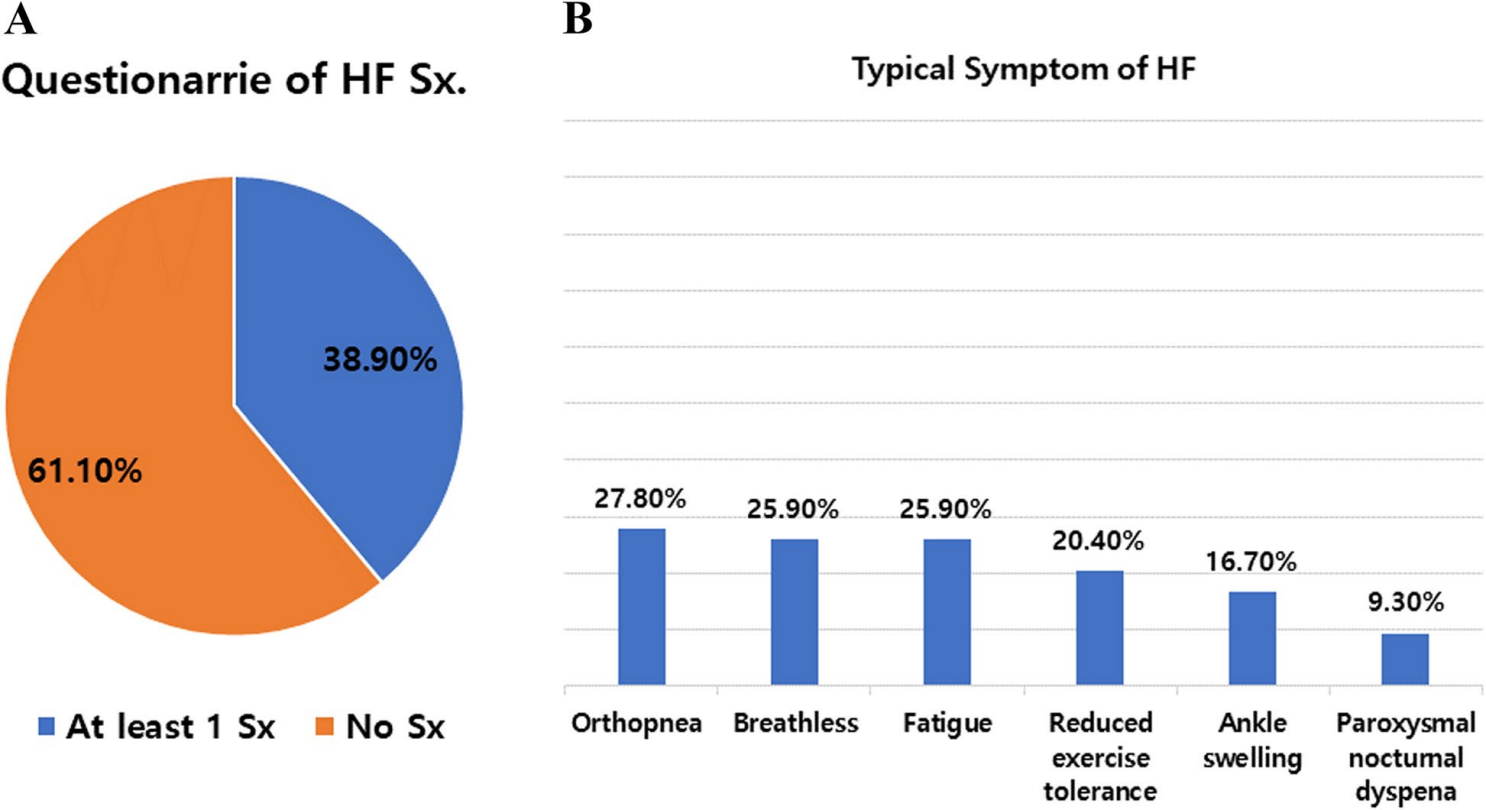
Questionnaire-based analysis to identify symptoms of HF by ESC guidelines. **A** Presence of HF symptoms. **B** Specific items of symptoms. ESC: European Society of Cardiology, HF: heart failure, Sx: symptom

Figure 1 shows the prevalence of structural or functional abnormalities in pre- and post-HD according to current HF guidelines (at least one of the following: LVMI ≥ 95 g/m^2^ in females, ≥ 115 g/m^2^ in males, LAVI > 34 mL/m^2^, E/e′ ratio > 14, TR jet velocity at rest > 2.8 m/s). A total of 88.8% of pre-HD and 66.6% of post-HD had structural or functional abnormalities by echocardiography (88.9 vs. 66.7%, *p* = 0.007). The percentage for each parameter is shown in Fig. 2. TR jet V frequency exceeded the abnormality criteria (> 2.8 m/s) pre- and post-HD 46% and 20% of the time, respectively, which was a large difference. The questionnaire-based analysis to identify symptoms and signs of HF by ESC guidelines is presented in Fig. 3A [8]. Twenty-one (38.9%) of the 54 patients had at least one symptom, and the remaining 33 (61.1%) did not show any symptom. The most frequent symptoms were orthopnea (27.8%), breathlessness (25.9%), and fatigue (25.9%) (Fig. 3B).

## Discussion

An important finding of our study is that patients undergoing HD may have different hemodynamic parameters depending on whether they are pre- or post-HD. In particular, we found significant differences in parameters of cardiac volume such as LVEDV, LVESD, and LAVI. These differences cause variance in the frequency of satisfying abnormality criteria of objective HF parameters. A total of 88.9% of patients immediately before HD and 66.7% immediately after HD had corresponding parameters; thus, there was an up to 22.2% difference between the time points. Medical records showed a weight change of about 2.8 kg after HD; we posit that this weight change is reflected in the echocardiographic parameters. Among echocardiographic parameters, TR jet V, RVSP, and LAVI showed remarkable changes according to volume difference, while EF, LV GLS, and E/E′ did not. Thus, TR jet V, RVSP, and LAVI seem to be dependent on volume status. The value of TR jet V showed a difference of 0.3 m/s between pre- and post-HD. This gap may not be large, but the difference in the frequency of satisfying the criteria of HF is more than twice due to this difference. TR jet V is an important parameter for HF diagnosis, but it is also important for predicting HF prognosis. For HFpEF, it is accepted that pulmonary vascular abnormalities lead to poor outcomes due to accompanying excessive right heart congestion and blunted right ventricular systolic reserves [15]. Our previous study suggested that there was a significant difference in TR jet V when echo was compared with and without acute decompensated HF for patients undergoing HD [16]. Therefore, diagnosing dialysis patients with only the measured parameters without considering when echocardiography was performed can yield diagnostic errors. Most hospitals in Korea do not have standards for when to perform echocardiography on dialysis patients, so establishing a common echocardiography protocol for dialysis patients will help reduce errors in diagnosing HF.

LAVI is a key determinant of diastolic function [12] and a predictor of adverse events of HF [17]. However, it varies greatly depending on patient volume at the time of echocardiography, and there may be errors depending on the examiner. Recently, there has been an attempt to utilize a parameter called LA reservoir strain to evaluate the volume and geometry of LA in more detail [18]. E/E′ is also a parameter that is significantly associated with LV filling pressure, and it can provide prognostic value in patients with ESRD [19]. The E/E′ cut-off value varies according to HF guidelines (ESC, ASE, and Korean Heart Failure Society). However, the significance of an E/E′ increase is clear; it is thought to be a reliable marker of increased LV filling pressure in ESRD the population [20]. In our results, E/E′ tended to be lower post-HD, but it was not statistically different from pre-HD (*p* = 0.299). The non-significance of our results could be due to low statistical power and a small number of enrolled patients.

It is not easy to diagnose HF dichotomously in clinical practice because it is based on comprehensive analysis of various indicators such as clinical presentation, echocardiographic parameters, and serum biomarkers. BNP and NT-proBNP levels are related to elevated LV filling pressure and play an important role in HF diagnosis [21]. However, as these levels are elevated in most patients with ESRD, there is a limit to their diagnostic use [22], and our data showed a similar trend. Most ESRD patients with one or more structural and functional abnormalities on echocardiography meet the diagnostic criteria for HF, as supported by our results. ESRD patients have more risk factors for HF than the general population, so a high prevalence among them is expected. Furthermore, most patients in our study had LVH and diastolic dysfunction. In our patients, while approximately 90% of patients showed objective abnormalities on pre-HD echocardiogram, approximately 60% of patients in our survey did not complain of symptoms of HF. After cardiac remodeling has progressed over long-term dialysis, many patients do well without decompensation, which presents a clinical dilemma about whether it is appropriate to diagnose these patients with HF. As a result, representative echocardiographic parameters may have limited application in ESRD patients because their cardiac structure and hemodynamics are different from those of the general population.

Our study had several limitations. First, this is a single-center study, and the number of enrolled patients was too small to establish a generalized conclusion. Second, this was not a blinded study. Third, there may be interobserver variability in echocardiography among examiners. However, to our knowledge, this is the first prospective study to evaluate hemodynamic parameter differences between pre- and post-HD and to apply recent HF guideline criteria for ESRD patients.

In conclusion, our data show that post-HD echocardiography showed significantly reduced LAVI, TR jet V, and RVSP compared with pre-HD. Most patients undergoing HD satisfy the diagnostic criteria for HF according to current HF guidelines, and there is a 22.2% difference when comparing patients before and after dialysis. There are limitations when applying the HF guidelines to dialysis patients, and caution is required.

## References

[1] Genovesi S, Valsecchi MG, Rossi E (2009). Sudden death and associated factors in a historical cohort of chronic haemodialysis patients. Nephrol Dial Transplant.

[2] Collins AJ, Foley RN, Chavers B (2012). United States Renal Data System 2011 Annual Data Report: Atlas of chronic kidney disease & end-stage renal disease in the United States. Am J Kidney Dis.

[3] Roehm B, Gulati G, Weiner DE (2020). Heart failure management in dialysis patients: many treatment options with no clear evidence. Semin Dial.

[4] Foley RN, Parfrey PS, Harnett JD (1995). Clinical and echocardiographic disease in patients starting end-stage renal disease therapy. Kidney Int.

[5] Agarwal R, Nissenson AR, Batlle D, Coyne DW, Trout JR, Warnock DG (2003). Prevalence, treatment, and control of hypertension in chronic hemodialysis patients in the United States. Am J Med.

[6] Chiu DY, Green D, Abidin N, Sinha S, Kalra PA (2014). Echocardiography in hemodialysis patients: uses and challenges. Am J Kidney Dis.

[7] Bossola M, Pepe G, Vulpio C (2018). The frustrating attempt to limit the interdialytic weight gain in patients on chronic hemodialysis: new insights into an old problem. J Ren Nutr.

[8] McDonagh TA, Metra M, Adamo M (2021). 2021 ESC Guidelines for the diagnosis and treatment of acute and chronic heart failure. Eur Heart J.

[9] Reddy YN, Carter RE, Obokata M, Redfield MM, Borlaug BA (2018). A simple, evidence-based approach to help guide diagnosis of heart failure with preserved ejection fraction. Circulation.

[10] Pieske B, Tschöpe C, de Boer RA (2020). How to diagnose heart failure with preserved ejection fraction: the HFA-PEFF diagnostic algorithm: a consensus recommendation from the Heart Failure Association (HFA) of the European Society of Cardiology (ESC). Eur J Heart Fail.

[11] Lang RM, Badano LP, Mor-Avi V (2015). Recommendations for cardiac chamber quantification by echocardiography in adults: an update from the American Society of Echocardiography and the European Association of Cardiovascular Imaging. J Am Soc Echocardiogr.

[12] Nagueh SF, Smiseth OA, Appleton CP (2016). Recommendations for the evaluation of left ventricular diastolic function by echocardiography: an update from the American Society of Echocardiography and the European Association of Cardiovascular Imaging. J Am Soc Echocardiogr.

[13] Rudski LG, Lai WW, Afilalo J (2010). Guidelines for the echocardiographic assessment of the right heart in adults: a report from the American Society of Echocardiography endorsed by the European Association of Echocardiography, a registered branch of the European Society of Cardiology, and the Canadian Society of Echocardiography. J Am Soc Echocardiogr.

[14] Silbiger JJ (2019). Pathophysiology and echocardiographic diagnosis of left ventricular diastolic dysfunction. J Am Soc Echocardiogr.

[15] Borlaug BA, Obokata M (2017). Is it time to recognize a new phenotype? Heart failure with preserved ejection fraction with pulmonary vascular disease. Eur Heart J.

[16] Kim BJ, Kim SJ, Im SI (2022). Increased right ventricular pressure as a predictor of acute decompensated heart failure in end-stage renal disease patients on maintenance hemodialysis. Int J Heart Fail.

[17] Leung DY, Boyd A, Ng AA, Chi C, Thomas L (2008). Echocardiographic evaluation of left atrial size and function: current understanding, pathophysiologic correlates, and prognostic implications. Am Heart J.

[18] Peters AC, Lee J, Jankowski M, Thomas JD (2023). Relationship between left atrial reservoir strain, volumes, and geometry: Insights from simple theoretical model. Echocardiography.

[19] Wang AY, Wang M, Lam CW, Chan IH, Zhang Y, Sanderson JE (2008). Left ventricular filling pressure by Doppler echocardiography in patients with end-stage renal disease. Hypertension.

[20] Sharma R, Pellerin D, Gaze DC (2006). Mitral peak Doppler E-wave to peak mitral annulus velocity ratio is an accurate estimate of left ventricular filling pressure and predicts mortality in end-stage renal disease. J Am Soc Echocardiogr.

[21] Svensson M, Gorst-Rasmussen A, Schmidt EB, Jorgensen KA, Christensen JH (2009). NT-pro-BNP is an independent predictor of mortality in patients with end-stage renal disease. Clin Nephrol.

[22] Schaub JA, Coca SG, Moledina DG, Gentry M, Testani JM, Parikh CR (2015). Amino-terminal pro-B-type natriuretic peptide for diagnosis and prognosis in patients with renal dysfunction: a systematic review and meta-analysis. JACC Heart Fail.

